# Random non-fasting C-peptide testing can identify patients with insulin-treated type 2 diabetes at high risk of hypoglycaemia

**DOI:** 10.1007/s00125-017-4449-2

**Published:** 2017-10-05

**Authors:** Suzy V. Hope, Bridget A. Knight, Beverley M. Shields, Anita V. Hill, Pratik Choudhary, W. David Strain, Timothy J. McDonald, Angus G. Jones

**Affiliations:** 1 NIHR Exeter Clinical Research Facility, University of Exeter Medical School and Royal Devon and Exeter NHS Foundation Trust, Barrack Road, Exeter, EX2 5DW UK; 2 Diabetes and Vascular Medicine, University of Exeter Medical School and Royal Devon and Exeter NHS Foundation Trust, Exeter, UK; 3 Department of Diabetes, King’s College London, London, UK

**Keywords:** Continuous glucose monitoring, C-peptide, Diabetes, Hypoglycaemia, Insulin, Type 2 diabetes

## Abstract

**Aims/hypothesis:**

The aim of this study was to determine whether random non-fasting C-peptide (rCP) measurement can be used to assess hypoglycaemia risk in insulin-treated type 2 diabetes.

**Methods:**

We compared continuous glucose monitoring-assessed SD of blood glucose and hypoglycaemia duration in 17 patients with insulin-treated type 2 diabetes and severe insulin deficiency (rCP *<* 200 pmol/l) and 17 matched insulin-treated control patients with type 2 diabetes but who had preserved endogenous insulin (rCP > 600 pmol/l). We then assessed the relationship between rCP and questionnaire-based measures of hypoglycaemia in 256 patients with insulin-treated type 2 diabetes and a comparison group of 209 individuals with type 1 diabetes.

**Results:**

Continuous glucose monitoring (CGM)-assessed glucose variability and hypoglycaemia was greater in individuals with rCP < 200 pmol/l despite similar mean glucose. In those with low vs high C-peptide, SD of glucose was 4.2 (95% CI 3.7, 4.6) vs 3.0 (2.6, 3.4) mmol/l (*p <* 0.001). In the low-C-peptide vs high-C-peptide group, the proportion of individuals experiencing sustained hypoglycaemia ≤ 4 mmol/l was 94% vs 41% (*p <* 0.001), the mean rate of hypoglycaemia was 5.5 (4.4, 6.7) vs 2.1 (1.4, 2.9) episodes per person per week (*p =* 0.004) and the mean duration was 630 (619, 643) vs 223 (216, 230) min per person per week (*p* = 0.01). Hypoglycaemia ≤ 3 mmol/l was infrequent in individuals with preserved C-peptide (1.8 [1.2, 2.6] episodes per person per week vs 0.4 [0.1, 0.8] episodes per person per week for low vs high C-peptide, *p =* 0.04) and only occurred at night. In a population-based cohort with insulin-treated type 2 diabetes, self-reported hypoglycaemia was twice as frequent in those with rCP < 200 pmol/l (OR 2.0, *p <* 0.001) and the rate of episodes resulting in loss of consciousness or seizure was five times higher (OR 5.0, *p =* 0.001). The relationship between self-reported hypoglycaemia and C-peptide was similar in individuals with type 1 and type 2 diabetes.

**Conclusions/interpretation:**

Low rCP is associated with increased glucose variability and hypoglycaemia in patients with insulin-treated type 2 diabetes and represents a practical, stable and inexpensive biomarker for assessment of hypoglycaemia risk.

**Electronic supplementary material:**

The online version of this article (10.1007/s00125-017-4449-2) contains peer-reviewed but unedited supplementary material, which is available to authorised users.

## Introduction

Individuals with type 1 diabetes usually develop severe endogenous insulin deficiency, which results in high glucose variability and hypoglycaemia risk [1–4]. Treatment guidelines for type 1 diabetes, therefore, incorporate early intensive strategies to minimise hypoglycaemia, including multiple daily insulin injections, carbohydrate counting and insulin pumps [5].

Endogenous insulin deficiency is best assessed using C-peptide [6]. In the DCCT study, intensively treated participants with type 1 diabetes with mixed meal test-stimulated C-peptide < 200 pmol/l had three times as many episodes of severe hypoglycaemia than other participants, despite having higher HbA_1c_ [2, 3]. This threshold of 200 pmol/l is commonly described as identifying absolute insulin deficiency, although modern assays can measure below this range [7, 8] and a relationship between hypoglycaemia and lower levels of C-peptide has been described [9, 10].

We have recently demonstrated that a random non-fasting plasma C-peptide (rCP) sample is a sensitive and specific measure for mixed meal tolerance test-defined absolute insulin deficiency (stimulated C-peptide < 200 pmol/l), with the same C-peptide threshold having a sensitivity and specificity of 100% and 93%, respectively [11]. As C-peptide is stable in whole blood collected into EDTA for > 24 h [12], this means severe insulin deficiency can be identified from a routine non-fasting blood sample when a patient is seen in the clinic.

Severe insulin deficiency can occur in insulin-treated patients who have clinical features consistent with type 2 diabetes, but the deficiency is usually not recognised [13–16]. This is likely to be an increasing problem as obesity rates increase, making clinical classification more difficult. We hypothesise that individuals with severe insulin deficiency will have high glycaemic variability and high hypoglycaemia risk whatever the underlying diabetes aetiology or classification. Identifying individuals with apparent type 2 diabetes who have developed severe insulin deficiency could assist appropriate management, including consideration of the intensive strategies to minimise hypoglycaemia traditionally used in type 1 diabetes.

We therefore aimed to determine whether rCP measurement can be used to assess risk of hypoglycaemia in insulin-treated patients with a clinical diagnosis of type 2 diabetes.

## Methods

We assessed whether severe insulin deficiency defined by a low rCP is associated with high rates of hypoglycaemia and glucose variability, as assessed by continuous glucose monitoring (CGM), in patients with insulin-treated type 2 diabetes. We replicated our findings using questionnaire-based assessment of hypoglycaemia in a large population cohort of patients with insulin-treated type 2 diabetes and a cohort with type 1 diabetes as a comparison group.

### Ethics approval

Ethics approvals were obtained from the National Research Ethics Service (NRES) Committee South West (UK).

### CGM assessment of glucose variability and hypoglycaemia in insulin-treated patients with type 2 diabetes with low or preserved endogenous insulin secretion

#### Participants

We recruited 17 insulin-treated individuals with a clinical diagnosis of type 2 diabetes and severe insulin deficiency and a control group of 17 matched individuals with relatively preserved endogenous insulin secretion. All participants were diagnosed aged 35 years or older, treated without insulin for at least 2 years beyond diagnosis and had an eGFR > 30 ml min^−1^ (1.73 m)^−2^. Participants were recruited based on known C-peptide result and clinical characteristics in the Diabetes Alliance for Research in England (DARE) study, http://www.diabetesgenes.org/content/diabetes-alliance-research-england-dare-previously-known-exeter-research-alliance-extra-stud, an unselected population-based study of adults with diabetes in Devon (UK), recruited predominantly through primary care. The 17 participants with severe insulin deficiency (rCP < 200 pmol/l) were individually matched by sex and HbA_1c_ (±10 mmol/l) with a control participant who had preserved endogenous insulin secretion (rCP *>* 600 pmol/l). C-peptide categories were chosen based on previously reported thresholds for severe insulin deficiency/high hypoglycaemia risk in type 1 diabetes (< 200 pmol/l) and for type 2 diabetes/insulin requirement (> 600 pmol/l) [6].

#### Baseline visit

Participants attended non-fasting within 5 h of a meal, without restriction on snacks or other intakes. Following informed consent, baseline characteristics were recorded and blood taken for repeat rCP, HbA_1c_ and islet autoantibody status (GAD/islet antigen 2 [IA2]). Clarke’s Hypoglycaemia Questionnaire [17] was completed by all participants.

#### CGM

At the baseline visit, participants commenced at least three consecutive days’ CGM (iPro2 Professional; Medtronic, Watford, UK). For calibration purposes, participants were asked to record four self-monitoring blood glucose tests daily over the CGM period.

The following criteria were required for CGM data to be included in analysis [18]: three or more self-monitoring of blood glucose (SMBG) calibrations in 24 h; no missing data points; correlation between SMBG and iPro2 readings > 0.77 in 24 h; mean difference between SMBG and iPro2 readings for each 24 h (MAD%) < 28% and a minimum of 24 h data meeting these criteria.

#### CGM analysis

The mean glucose, SD of glucose measurements, mean amplitude of glycaemic excursions (MAGE) and low blood glucose index (LBGI) were analysed for each individual using EasyGV online software [19], N. Hill, University of Oxford, Oxford, UK; https://www.phc.ox.ac.uk/research/technology-outputs/easygv.

An episode of hypoglycaemia was defined as ≥ 20 min at or below the interstitial glucose level of 4, 3 or 2.2 mmol/l, and only complete once readings had been above the threshold for > 20 min [19]. Results were converted to rates of hypoglycaemia and duration of hypoglycaemia per person per week, and by day (08:00–00:00 hours) and night (00:00–08:00 hours).

#### Laboratory analyses

C-peptide was analysed using the automated Roche diagnostics (Manheim, Germany) E170 immuno-analyser (limit of detection 3.3 pmol/l, inter- and intra-assay coefficients of variation < 4.5% and < 3.3%, respectively) in the Blood Sciences department, Royal Devon and Exeter Hospital.

GAD65 and IA2 autoantibodies were assessed using the RSR Elisa kits (Cardiff, UK) on a Dynex DS2 automated Elisa System (Dynex Technologies, Worthing, UK). Cut-offs used were based on the 97.5th centile for 1600 adults without diabetes; the reference positive value was > 11 U/ml for GAD65 and > 15 U/ml for IA2.

#### Statistical analysis

Differences in continuous measures of glucose variability and hypoglycaemia and baseline characteristics between low- and high-C-peptide pairs were assessed using paired *t* tests, as differences in paired data approximated normal distribution. For comparing proportions, χ^2^ tests were used.

To assess whether our findings were independent of insulin regime, a potential source of confounding [20, 21], we performed an additional unpaired sensitivity analysis comparing measures of glucose variability and hypoglycaemia between C-peptide groups, with adjustment for use of prandial or mixed insulin using ANCOVA and (for rates) Poisson regression.

### Assessment of the relationship between rCP and self-reported hypoglycaemia in patients with insulin-treated diabetes

#### Participants

Four hundred and sixty-five (256 type 2 diabetes, 209 type 1 diabetes, reported clinician diagnosis) insulin-treated patients in the DARE study (see above), were recruited by postal invitation (existing DARE cohort) or at DARE recruitment visit, of whom 27 also participated in our CGM cohort (above).

#### C-peptide assessment

Non-fasting C-peptide was measured at participants’ recruitment visit or, for participants previously recruited to the DARE study, with prior written informed consent, measured on residual plasma from routine non-fasting clinical laboratory analysis.

#### Hypoglycaemia questionnaires

Participants completed a modified Clarke’s Hypoglycaemia Questionnaire [17, 22] by post (existing DARE cohort) or at DARE recruitment visit. Hypoglycaemia awareness was assessed by calculating the Clarke score [17]: questions are each allocated a score of 0 (aware) or 1 (reduced awareness), and the score for the seven questions added together. A score of 4 or more is classed as ‘reduced awareness’, as previously reported [17].

Clarke’s Hypoglycaemia question 5 (frequency of episodes in the last month where blood glucose < 3.5 mmol/l, with symptoms) and question 6 (episodes where blood glucose < 3.5 mmol/l, without symptoms) categorise self-reported frequency into groups. Thus, for analysis we assigned an estimated frequency per answer for an approximation of frequency in the last month: 1–3 episodes in the last month became 2; one episode per week became 1 × 4 = 4; two or three episodes per week became 2.5 × 4 = 10; four or five episodes per week became 4.5 × 4 = 18 and almost daily was estimated at 25 episodes per month.

#### Statistical analysis

Rates of self-reported hypoglycaemia were assumed to follow a Poisson distribution, so data are presented as incidence rates and incidence rate ratios. We compared rates of self-reported hypoglycaemia < 3.5 mmol/l (Clarke questions 5 and 6, events per person per month) in individuals with a clinical diagnosis of type 2 diabetes with and without rCP < 200 pmol/l using the *z* test. CIs around rates were calculated using the Poisson distribution. We compared the proportion of participants reporting at least one episode of hypoglycaemia in Clarke questions 3–6, and the proportion with calculated reduced hypoglycaemia awareness (Clarke score ≥ 4), using the χ^2^ test.

To assess whether differences between groups were due to confounding by differences in clinical features associated with hypoglycaemia and insulin type [21], we assessed the OR (proportions, logistic regression) and rate ratio (questions 5 and 6 rate, Poisson regression) for the above outcomes with and without adjustment for age, sex, HbA_1c_ and use of prandial or mixed insulin (vs intermediate- or long-acting insulin only). These covariates were associated with self-reported hypoglycaemia rate in univariate analysis (*p <* 0.002 for all).

We repeated this analysis in individuals with type 1 diabetes.

To ensure results did not reflect poor accuracy of reported clinical diagnosis, we also repeated analysis in subgroups defined by the following restrictive criteria for type 1 and 2 diabetes: (1) type 1 diabetes–clinician diagnosis of type 1 diabetes, diabetes onset before age 35 years and insulin treatment within 6 months of diagnosis and (2) type 2 diabetes–clinician diagnosis of type 2 diabetes, diagnosis ≥ age 35 years and time to insulin ≥ 2 years.

## Results

### Assessment of glucose variability and hypoglycaemia in insulin-treated patients with type 2 diabetes with low or preserved endogenous insulin secretion

#### Participant characteristics and data quality

Participant characteristics are shown in Table 1. Mean CGM glucose (10.2 vs 9.9 mmol/l, *p =* 0.5), HbA_1c_, age, duration of diabetes and BMI were similar when comparing the low- and high-C-peptide groups. However, participants with severe insulin deficiency had progressed more rapidly to insulin treatment, received higher insulin doses and were more likely to receive basal bolus insulin therapy and have positive islet cell autoantibodies.

**Table 1:** Characteristics of the CGM-assessed hypoglycaemia cohort

Characteristic	C-peptide < 200 pmol/l	C-peptide > 600 pmol/l	*p* value
No. of participants	17	17	–
C-peptide, pmol/l	38.9 (10.3, 67.4)	1238.3 (906.5, 1570.1)	–
Mean glucose on CGM, mmol/l	10.2 (9.1, 11.3)	9.9 (8.6, 11.1)	0.50
HbA_1c_, mmol/mol	72.0 (65.5, 78.5)	72.2 (66.1, 78.3)	0.88
HbA_1c_, %	8.7 (8.1, 9.3)	8.7 (8.4, 9.3)	0.88
Male sex, *n* (%)	11 (65)	11 (65)	–
Age, years	72.8 (68.4, 77.2)	71.8 (68.5, 75.1)	0.71
Diabetes duration, years	24.5 (19.0, 30.0)	19.8 (16.8, 22.9)	0.13
BMI, kg/m^2^	26.6 (24.8, 28.4)	27.9 (26.1, 29.8)	0.19
Time to insulin, months	62 (37, 87)	111 (76, 145)	0.03
Total dose of insulin in 24 h, U/kg	0.71 (0.61, 0.8)	0.52 (0.39, 0.64)	0.007
Use of prandial (basal bolus or mixed) insulin, %	100	59	0.003
Proportion with ≥ one islet autoantibody, %	59 (28, 100)	6 (2, 33)	<0.001

Values reported are mean (95% CI) unless stated otherwise

The mean duration of CGM recording meeting inclusion criteria for analysis was 4.1 (range 1–6.2) days; this was similar between the two groups (4.3 [range 1–6.2] vs 3.9 [1.3–6] days in the low- vs high-C-peptide group, respectively, *p =* 0.34).

#### SD of glucose readings on CGM was higher in the low-C-peptide group

Glucose variability was greater in the low-C-peptide group: SD of glucose measurements 4.15 mmol/l (95% CI 3.67, 4.64) vs 3.01 mmol/l (2.65, 3.38), *p <* 0.001. MAGE did not differ between groups (7.05 mmol/l [5.9, 8.2] vs 6.03 mmol/l [4.8, 7.3], *p =* 0.1).

#### Hypoglycaemia is markedly more frequent in individuals with low C-peptide

**Fig. 1:**
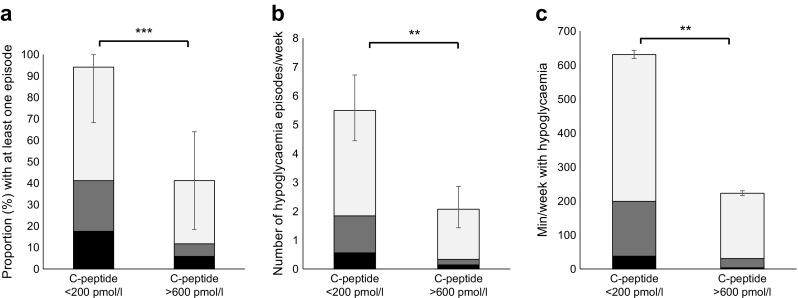
Hypoglycaemia measured by CGM in individuals with rCP < 200 pmol/l vs > 600 pmol/l. Light grey section of bars, glucose threshold ≤ 4 mmol/l; dark grey sections, ≤ 3 mmol/l; black sections, ≤ 2.2 mmol/l. Error bars represent 95% CI. (**a**) Proportion of individuals with one or more hypoglycaemia episodes. (**b**) Rate of hypoglycaemia (no. of episodes per person per week). (**c**) Hypoglycaemia duration (minutes per person per week). **p≤0.01 and ***p<0.001 for rCP <200 pmol/l vs >600 pmol/l

Of the 17 participants in the low-C-peptide group, 16 (94%) experienced at least one episode of hypoglycaemia on CGM (≥ 20 min, ≤ 4 mmol/l) compared with 7/17 (41%) in the high-C-peptide group (*p <* 0.001) (Fig. 1a).

The rate of hypoglycaemia (≥ 20 min, ≤ 4 mmol/l) on CGM was higher in the low-C-peptide group than in the high-C-peptide group (5.5 [95% CI 4.4, 6.7] vs 2.1 [1.4, 2.9] episodes per person per week, *p* = 0.004) (Fig. 1b). The total duration of hypoglycaemia was also higher in the low-C-peptide group (mean 630 [95% CI 329, 931] vs 223 [14, 431] min per person per week, *p* = 0.01) (Fig. 1c).

The frequency and total duration of episodes of more significant hypoglycaemia (interstitial glucose ≤ 3 mmol/l) were also higher in the low-C-peptide group (1.8 [95% CI 1.2, 2.6] vs 0.4 (0.1, 0.8) episodes per person per week, *p =* 0.037; 199 (47, 352) vs 31 [0, 83] min per person per week, *p =* 0.049) (Fig. 1b, c). All hypoglycaemia episodes below 3 mmol/l in the high-C-peptide group occurred at night (00:00–08:00 hours); in contrast 7/17 (41%) of participants with low C-peptide experienced daytime hypoglycaemia ≤3 mmol/l, with a mean rate of 1.3 (0.8, 2.0) episodes per person per week (*p =* 0.01). Rates of severe hypoglycaemia ≤ 2.2 mmol/l were low and did not significantly differ by C-peptide status (0.5 [0.2, 1] vs 0.1 [0,0.4] episodes per person per week, *p =* 0.26) (Fig. 1b). The LBGI was substantially higher in those with low C-peptide (5.5 [3.8, 7.3] vs 1.9 [0.8, 3.0], *p <* 0.001).

#### Associations between C-peptide and hypoglycaemia were independent of insulin regimen

In unpaired sensitivity analysis, the association between C-peptide group and both glucose variability and hypoglycaemia was not substantially altered by adjustment for insulin regimen (see electronic supplementary material [ESM] Tables 1 and 2).

### Replication using self-reported hypoglycaemia in a population cohort

#### Participant characteristics

Participant characteristics for the hypoglycaemia questionnaire cohort are shown in Table 2 (type 2 diabetes) and ESM Table 3 (type 1 diabetes comparison cohort). Fourteen per cent of participants with a clinical diagnosis of insulin-treated type 2 diabetes had rCP < 200 pmol/l. These participants had similar age, diabetes duration and HbA_1c_ to participants with retained C-peptide but had lower BMI and time to insulin and greater use of prandial insulin.

**Table 2:** Characteristics of the questionnaire-assessed hypoglycaemia cohort with insulin-treated type 2 diabetes

Characteristics	C-peptide < 200 pmol/l	C-peptide ≥ 200 pmol/l	*p* value
No. (%) of participants	35 (14)	221 (86)	–
HbA_1c_, mmol/mol	71.3 (66.0, 76.6)	66.9 (64.7, 69.1)	0.1
HbA_1c_, %	8.7 (8.2, 9.2)	8.3 (8.1, 8.5)	0.1
Male sex, %	60 (42, 76)	63 (56, 69)	0.7
Age, years	68.2 (64.4, 72.0)	66.0 (64.7, 73)	0.2
Duration of diabetes, years	16.2 (13.1, 19.5)	14.1 (13.1, 15.2)	0.2
BMI, kg/m^2^	28.2 (26.4, 30.0)	32.2 (31.5, 33.0)	<0.001
Time to insulin, months	48.4 (28.4, 68.5)	84.1 (73.2, 95.0)	0.01
Insulin dose in 24 h, U/kg	0.73 (0.58, 0.87)	0.63 (0.56, 0.69)	0.3
Use of prandial (basal bolus or mixed) insulin, %	84 (64, 95)	44 (36, 51)	<0.001

Values are reported as mean (95% CI) unless stated otherwise

#### Low C-peptide is associated with higher rates of self-reported hypoglycaemia in patients with insulin-treated type 2 diabetes

**Fig. 2:**
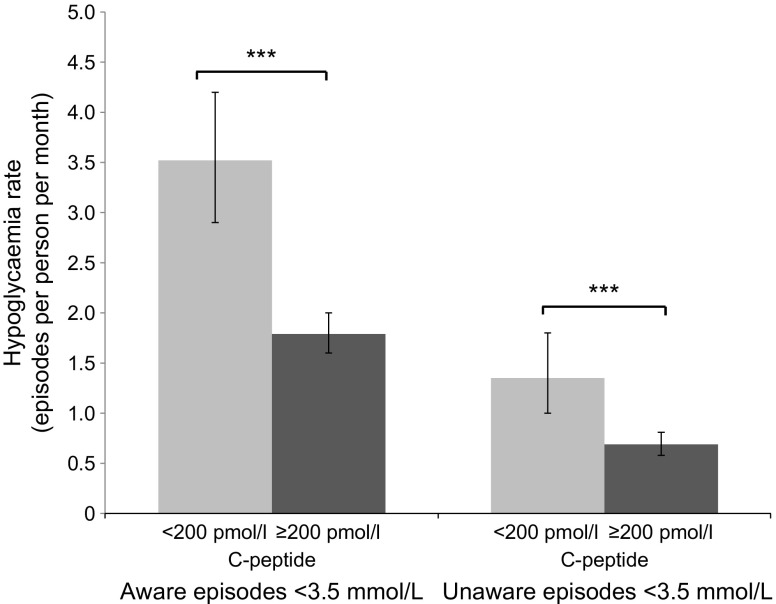
Frequency of self-reported hypoglycaemia (< 3.5 mmol/l) by C-peptide status in patients with insulin-treated type 2 diabetes (*n*=256). Light grey bars, rCP < 200 pmol/l; dark grey bars, rCP ≥ 200 pmol/l. Rates of aware and unaware episodes derived from Clarke questions 5 and 6, respectively. Error bars represent 95% CI. ***p<0.001 for rCP < 200 pmol/l vs ≥ 200 pmol/l

In participants with insulin-treated type 2 diabetes, self-reported hypoglycaemia was approximately twice as common in those with rCP < 200 pmol/l compared with those with rCP ≥ 200 pmol/l (Fig. 2). The mean (95% CI) rate of occurrence of symptoms (Clarke question 5) in low- vs high-C-peptide participants was 3.5 (2.9, 4.2) vs 1.8 (1.6, 2.0) episodes per person per month (*p <* 0.001). The mean rate without symptoms (Clarke question 6) was 1.4 (1.0,1.8) vs 0.69 (0.58, 0.81) episodes per person per month (*p <* 0.001) in the low- vs high-C-peptide group. Severe hypoglycaemia resulting in unconsciousness or seizures (Clarke question 4) was more common in those with low C-peptide (OR 5.0 [95% CI 2.0, 12.7]) but the frequency of episodes needing external help (Clarke question 3) was not different (*p =* 0.5). ORs and rate ratios for self-reported hypoglycaemia (Clarke questions 3–6), by C-peptide status, are given in ESM Table 4, without and with adjustment for prandial insulin use, age, sex and HbA_1c_. Results were not substantially altered by adjusting for these covariates.

Questionnaire-defined hypoglycaemia unawareness (Clarke score ≥ 4) occurred in 11.4% and 5.0% of those with low and high C-peptide, respectively; the difference was statistically significant only in adjusted analysis (unadjusted *p =* 0.17, adjusted *p =* 0.005) (ESM Table 4).

#### The relationship between self-reported hypoglycaemia and C-peptide is similar in type 1 and type 2 diabetes

In participants with type 1 diabetes, rCP < 200 pmol/l was also associated with an approximate doubling of rate of self-reported hypoglycaemia in the previous month with or without symptoms: mean (95% CI) rate with symptoms (Clarke question 5) (low vs high C-peptide) 6.3 (6.0, 6.7) vs 3.0 (2.4, 3.7) episodes per person per month (*p <* 0.001); without symptoms (question 6) 1.7 (1.5, 1.9) vs 0.6 (0.36, 0.98) episodes per person per month (*p <* 0.001). The difference in hypoglycaemia unawareness (Clarke score ≥ 4) was not statistically different between C-peptide groups (11.7% [95% CI 7.4, 17.4] and 3.3% [0, 17.2] for those with C-peptide < 200 pmol/l and ≥ 200 pmol/l, respectively, *p =* 0.2). Results were similar after adjustment for clinical features and prandial insulin (ESM Table 4).

#### Thirteen per cent of patients with insulin-treated type 2 diabetes have severe insulin deficiency, even when defined by more strict criteria

When using strict criteria for type 2 diabetes (clinician diagnosis of type 2 diabetes, age at diagnosis ≥ 35 years and insulin initiation ≥ 2 years from diagnosis, *n* = 203), 13% (95% CI 8.5, 18) of individuals meeting these criteria had C-peptide < 200 pmol/l. When restricting analysis to this cohort, results were similar, with higher rates of hypoglycaemia in those with low vs high C-peptide (Clarke question 5, 3.0 vs 1.9 episodes per person per month [*p <* 0.001]; Clarke question 6, 1.6 vs 0.5 episodes per person per month [*p <* 0.001]). Results were similar for participants meeting strict criteria for type 1 diabetes (*n* = 188), of whom 12% had rCP ≥ 200 pmol/l (Clarke question 5, 6.1 vs 3.6 episodes per person per month [*p <* 0.001]; Clarke question 6, 1.8 vs 0.7 episodes per person per month [*p <* 0.001]).

#### Hypoglycaemia rates in type 1 and type 2 diabetes with low insulin secretion are broadly similar after accounting for clinical features and insulin type

In unadjusted analysis, type 1 diabetes with C-peptide < 200 pmol/l was associated with a 1.7 times higher rate of hypoglycaemia than the rate reported by participants with type 2 diabetes and C-peptide < 200 pmol/l: rate ratio for episodes of hypoglycaemia < 3.5 mmol/l in the previous month in those with rCP < 200 pmol/l for type 1 vs type 2 diabetes (Clarke questions 5 and 6 combined) was 1.66 (95% CI 1.39,1.97) (*p <* 0.001). However, after adjustment for prandial insulin use, HbA_1c_, sex and age this difference was modest and did not reach statistical significance (rate ratio 1.18 [0.96, 1.45], *p =* 0.12).

## Discussion

Our results demonstrate that patients with insulin-treated type 2 diabetes but low C-peptide levels have markedly increased incidence of hypoglycaemia in comparison to those with retained C-peptide, whether measured by CGM or self-reported. On CGM, glycaemic variability (when defined by SD, the most robust measure [19, 23–25]), frequency and duration of hypoglycaemia were markedly increased in those with severe insulin deficiency. More significant daytime hypoglycaemia (< 3 mmol/l, [26]) was entirely confined to these participants. These differences occurred despite similar glycaemic control in those with and without preserved endogenous insulin secretion. Severe insulin deficiency was not uncommon in participants with insulin-treated type 2 diabetes in our cohort, occurring in 13% of participants even when strict clinical criteria for classification were applied. While many of these participants may have diabetes of autoimmune aetiology, this was not clinically recognised or apparent, with low-C-peptide participants having late-onset diabetes, raised BMI and time from diagnosis to insulin treatment of several years.

### Comparison with other studies

Our findings of a strong association between C-peptide and hypoglycaemia are consistent with previous findings in type 1 diabetes using self-reported hypoglycaemia [1, 3, 9, 10, 19] and CGM studies in type 1 diabetes demonstrating higher SD or CV (SD/glucose), defined glucose variability and hypoglycaemia in individuals with lower C-peptide, including after islet transplant [27–29]. In these studies, C-peptide was measured using a mixed meal tolerance test, which is not suitable for routine use in clinical practice. One study examined the correlation between fasting C-peptide and glucose variability (CGM SD) and found inverse associations in both type 1 diabetes and insulin-treated type 2 diabetes, although hypoglycaemia was not examined [30].

The relationship between C-peptide levels and frequency of hypoglycaemia in those with a clinical diagnosis of type 2 diabetes is less widely recognised. The frequency of hypoglycaemia in type 2 diabetes is known to be associated with diabetes duration [31], and is markedly higher in individuals treated with insulin for a long period than in those in the initial stages of treatment [32]. It has also recently been shown that individuals with type 2 diabetes who were unable to achieve the ACCORD study’s treatment target of HbA_1c_ < 6.0% (42 mmol/mol) due to severe hypoglycaemia had lower C-peptide, with an OR of 23 for low C-peptide (baseline fasting C-peptide < 0.15 nmol/l) [33].

While the potential utility of rCP as a biomarker for hypoglycaemia in individuals with diabetes has not been previously examined, previous research has shown that this test is highly correlated with mixed-meal-test-measured C-peptide [11] and has similar or superior utility to glucagon-stimulated C-peptide assessment for differentiating between type 1 and type 2 diabetes [34].

### Strengths and limitations

This is to our knowledge the first study to examine the relationship between C-peptide and CGM-assessed hypoglycaemia in type 2 diabetes and the first to assess the potential utility of rCP for stratification of hypoglycaemia risk. This is an inexpensive test that can be measured at the point of clinical contact and could therefore easily be incorporated into clinical practice. Our additional study in a larger cohort using self-reported hypoglycaemia as an outcome strengthens our findings. We have assessed an older adult population in our study, an age group wherein hypoglycaemia is often not recognised, and consequences are more severe [35] and where risk biomarker-based stratification would therefore be particularly helpful.

A weakness of our study is that C-peptide assessment in our replication study was in the most part performed on routine non-fasting plasma–EDTA samples received by our laboratory, therefore did not assess concurrent glucose or timing of samples in relation to meals. We have previously shown concurrent hypoglycaemia may result in reduced rCP levels and recommended that when assessing rCP, concurrent hypoglycaemia is excluded and a sample taken 1–5 h post meal [11]. An additional weakness is that we cannot fully account for potential confounders which may alter the relationship between C-peptide and hypoglycaemia. In our CGM study, although most clinical characteristics were very similar between groups, prandial insulin use was more common in those with low C-peptide. This may reflect a causal association (those with low C-peptide and high glucose variability are likely to need prandial insulin for glycaemic control), although insulin regimen may directly influence hypoglycaemia [20, 21]. Importantly, adjusting for insulin regime in our analysis did not alter our findings. In our questionnaire study, rates of self-reported hypoglycaemia were likely to be very dependent on rates of self-monitoring of blood glucose levels, which was not assessed.

### Clinical implications

Our results suggest that an rCP sample can identify patients with insulin-treated type 2 diabetes who have a markedly increased risk of hypoglycaemia. These patients could not be identified by their clinical characteristics and only 59% would be identified by islet autoantibody testing, supporting the potential utility of rCP testing, which is practical, stable and inexpensive and therefore ideal for clinical use.

Identifying patients with insulin-treated type 2 diabetes at high risk of hypoglycaemia would be clinically helpful in guiding management (e.g. in setting appropriate glycaemic targets and levels of glucose monitoring) and would potentially allow consideration of treatment strategies for high glucose variability traditionally used in type 1 diabetes, such as carbohydrate counting and use of subcutaneous insulin pumps. An additional area where a robust, easily measurable biomarker for hypoglycaemia risk would be useful is in stratification of hypoglycaemia risk in relation to driving. Our CGM data showing no daytime episodes ≤ 3 mmol/mol in those with high rCP and an OR of 9.5 for self-reported severe hypoglycaemia (unconscious or fit, adjusted analysis) in the last year in those with low rCP are of interest in this regard and warrant further investigation.

### Unanswered questions and future research

In this study, we used a previously defined threshold for hypoglycaemia risk based on previous studies in type 1 diabetes, and in our CGM cohort we excluded participants with C-peptide between 200 and 600 pmol/l (23% and 7% of participants in our type 2 diabetes and type 1 diabetes questionnaire cohorts, respectively). The relationship between C-peptide and glucose variability in previous literature is continuous but non-linear, with a very strong association at low but not high C-peptide levels [28, 29, 36]. This is similar to the relationship observed with self-reported hypoglycaemia in our cohort (ESM Fig. 1). Further studies are needed to define optimal clinical cut-offs in obese populations with type 2 diabetes or alternatively to examine more complex approaches to risk prediction which might account for C-peptide as a continuous variable alongside other predictive features. Larger studies are needed to assess rCP against robust clinically important hypoglycaemia outcomes such as severe hypoglycaemia and to formally assess effectiveness and cost effectiveness of biomarker-based stratification of management. While it is likely that our results will be equally applicable to individuals diagnosed with type 1 diabetes, we did not directly assess this in our CGM study, which is thus an area of future work.

## Conclusions

Low rCP is associated with increased glucose variability and hypoglycaemia in patients with insulin-treated type 2 diabetes and represents a practical, stable and inexpensive biomarker for assessment of hypoglycaemia risk.

## Electronic supplementary material

ESM (PDF 315 kb)

## Abbreviations

CGM: Continuous glucose monitoring
DARE: Diabetes Alliance for Research in England
IA2: Islet antigen 2
LBGI: Low blood glucose index
MAGE: Mean amplitude of glycaemic excursions
rCP: Random non-fasting C-peptide
SMBG: Self-monitoring of blood glucose
