# Supplementary material for: Random non-fasting C-peptide testing can identify patients with insulin-treated type 2 diabetes at high risk of hypoglycaemia
Source: Diabetologia. 2017 Oct 5;61(1):66–74. doi: 10.1007/s00125-017-4449-2 (PMC6002965; doi:10.1007/s00125-017-4449-2)
Supplement: Supplementary file 1 — (PDF 315 kb) [file 125_2017_4449_MOESM1_ESM.pdf]

Electronic Supplementary Material for "Random non-fasting C-peptide testing can identify patients with insulin-treated type 2 diabetes at high risk of hypoglycaemia"

	C-peptide <200pmol/L	C-peptide ≥600pmol/L	p
Glucose SD (mmol/L)	4.1 (3.7, 4.5)	3.1(2.7, 3.4)	<0.001
MAGE	7.2 (6.0, 8.4)	5.9 (4.6, 7.1)	0.1
Duration ≤4.0 mmol/L (minutes per week)	631 (405, 855)	222 (0, 448)	0.02
Duration ≤3.0 mmol/L (minutes per week)	184 (66, 302)	46 (0, 164)	0.13
Duration ≤2.2 mmol/L (minutes per week)	37 (2, 71)	5 (0, 40)	0.2
LBGI	5.5 (3.8, 7.3)	1.9 (0.8, 3.0)	< 0.001

ESM Table 1: CGM mean (95% CI) glucose variability (SD, MAGE), hypoglycaemia duration and Low Blood Glucose Index (LBGI) in those with C-peptide <200pmol/L and >600pmol/L, adjusted for Mean glucose and use of prandial insulin (ANCOVA). N=34

Characteristic	Rate ratio (95% CI)	p	Adjusted rate ratio (95% CI)	p
Rate ≤4.0 mmol/L (episodes per week)	2.7 (1.8, 3.9)	<0.001	3.5 (2.0, 6.2)	<0.001
Rate ≤3.0 mmol/L (episodes per week)	5.5 (2.3, 13.6)	<0.001	3.0 (1.2, 7.4)	0.015
Rate ≤2.2 mmol/L (episodes per week)	6.0 (0.7, 49.8)	0.10	3.5 (0.4, 29.5)	0.2

ESM Table 2: Rate ratios for CGM-detected hypoglycaemia in those with C-peptide <200pmol/L (versus >600pmol/L), unadjusted and adjusted for HbA1c and prandial insulin (Poisson regression). N=34

	C-peptide <200pmol/L	C-peptide ≥200pmol/L	p
N	179 (86%)	30 (14%)	
Age (years)	50.1 (48.5, 52.8)	55.1 (49.4, 60.8)	0.1
Age of Diagnosis (years)	25.4 (23.1, 27.8)	44.7 (38.7, 50.6)	<0.0001
Duration diabetes (years)	25.4 (23.0, 27.5)	10.4 (6.7, 14.1)	<0.0001
BMI (kg/m)	26.7 (26.0, 27.4)	27.2 (25.1, 29.3)	0.6
% Male	46	40	0.7
HbA1c (mmol/mol)	66.8 (64.8, 68.9)	63.4 (55.0, 71.8)	0.2
HbA1c (%)	8.3 (8.1,8.5)	8.0 (7.2, 8.7)	0.2
Time to insulin (months)	0.6 (0.4, 0.8)	1.5 (0.2, 2.8)	<0.001
Insulin dose (u/KG)	0.62 (0.57, 0.67)	0.60 (0.40, 0.80)	0.7
Use of prandial or mixed insulin (%)	98 (94, 100)	72 (51, 88)	<0.0001

ESM Table 3: Characteristics of participants with a clinical diagnosis of Type 1 diabetes in the hypoglycaemia questionnaire cohort, by C-peptide status. Mean (95% confidence interval)

Characteristic	Odds ratio or rate ratio (95% CI)	p	Adjusted odds or rate ratio (95% CI)	p
Q3: 1 or more hypoglycaemic episodes needing external help in the last 6 months	1.3 (0.6, 2.7)	0.5	1.5 (0.6, 3.8)	0.4
Q4: 1 or more severe hypoglycaemia episodes (unconscious/seizure) in the last year	5.0 (2.0, 12.7)	0.001	9.5 (2.6, 33.8)	<0.001
Q5: 1 or more hypoglycaemia episodes in the last month with symptoms	2.1 (1.0, 4.6)	0.06	1.9 (0.7, 5.1)	0.2
Q5 hypoglycaemia rate: episodes with symptoms, per person per month	2.0 (1.6, 2.4)	<0.001	1.6 (1.2, 2.1)	<0.001
Q6: 1 or more hypoglycaemia episodes in the last month without symptoms	2.1 (0.90, 5.0)	0.09	2.1 (0.7, 6.5)	0.2
Q6 Rate: hypoglycaemia rate: episodes without symptoms, per person per month	2.0 (1.4, 2.8)	<0.001	4.1 (2.7, 6.1)	<0.001
Hypoglycaemia unaware (Clarke score >=4)	2.5 (0.74, 8.2)	0.17	12.1	0.005

ESM Table 4a: Type 2 diabetes - odds ratios and rate ratios for those with C-peptide <200pmol/L versus ≥200pmol/L for self-reported hypoglycaemia in the Clarke Questionnaire (Questions 3 to 6), unadjusted and adjusted for age, gender, HbA1c and prandial insulin.

Characteristic	Odds ratio or IRR 95% CI	p	Adjusted odds ratio/IRR	p
Q3: 1 or more episodes needing external help in the last 6 months	1.2 (0.53, 2.8)	0.6	1.2 (0.4,3.3)	0.7
Q4 1 or more severe hypoglycaemia episodes (unconscious/seizure) in the last year	1.2 (0.3, 4.3)	0.8	3.2 (0.5, 19.7)	0.2
Q5: 1 or more hypoglycaemia episodes in the last month with symptoms	6.8 (2.6, 17.6)	<0.001	7.1 (2.3, 22.1)	0.001
Q5 hypoglycaemia rate: episodes with symptoms, per person per month	2.1 (1.7, 2.6)	<0.001	2.0 (1.6, 2.6)	<0.001
Q6: 1 or more hypoglycaemia episodes in the last month without symptoms	2.6 (0.94, 7.1)	0.07	2.1 (0.68, 6.5)	0.2
Q6 Rate: hypoglycaemia rate: episodes without symptoms, per person per month	2.7 (1.7, 4.4)	<0.001	2.0 (1.2, 3.2)	0.008
Hypoglycaemia unaware (Clarke score >=4)	3.9 (0.50, 29.8)	0.12	4.3 (0.55, 33.5)	0.17

ESM Table 4b: Type 1 diabetes - odds ratios and rate ratios for those with C-peptide <200pmol/L versus ≥200pmol/L for self-reported hypoglycaemia in the Clarke Questionnaire (Questions 3 to 6), unadjusted and adjusted for age, gender, HbA1c and prandial insulin.

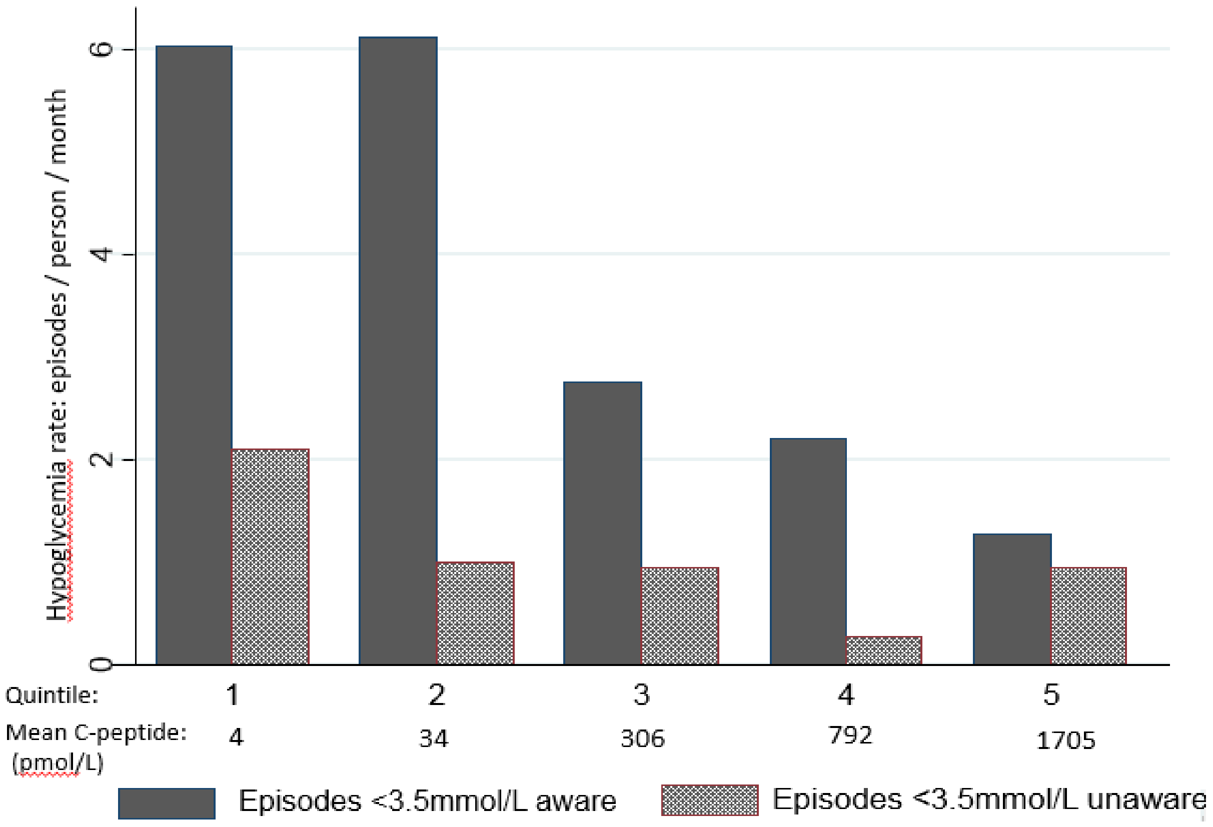

ESM Fig. 1: Rate of hypoglycaemia (self-reported episodes blood glucose <3.5mmol/mol) in the last month (Clarke's Hypoglycaemia Q5 & 6) shown by quintiles of C-peptide. Whole cohort (n=465). Mean C-peptide per quintile shown below.
